# Copper(II) Sulfamate: An Efficient Catalyst for the One-Pot Synthesis of 3,4-Dihydropyrimidine-2(1*H*)-ones and thiones

**DOI:** 10.3390/molecules14020763

**Published:** 2009-02-13

**Authors:** Chen-Jiang Liu, Ji-De Wang

**Affiliations:** 1 School of Science, Xi'an Jiaotong University, 710049 Xi'an, P. R. China; E-mail: pxylcj@126.com (C-J.L.); 2 School of Chemistry and Chemical Engineering, Xinjiang University, Key Laboratory of Oil and Gas Fine Chemicals, Ministry of Education, 830046 Urumqi, P. R. China

**Keywords:** Copper(II) sulfamate, Biginelli reaction, Dihydropyrimidinones.

## Abstract

A simple, efficient procedure for the one-pot Biginelli condensation reaction of aldehydes, *β*-ketoesters and urea or thiourea employing copper(II) sulfamate as a novel catalyst is described. Compared to the classical Biginelli reaction conditions, the present method has the advantages of good yields, short reaction times and experimental simplicity.

## Introduction

Dihydropyrimidinones (DHPMs) and their derivatives are well known heterocyclic units in the realm of natural and synthetic organic chemistry due to their wide spectrum of biological and therapeutic properties such as antibacterial, antiviral, antitumor and anti-inflammatory activities [1,2,3]. Recently, appropriately functionalized DHPM analogs have emerged as integral backbones of several calcium channel blockers, antihypertensive agents and α-la-adrenergic receptor antagonists [4,5]. Moreover, several alkaloids containing the dihydropyrimidine core unit that have been isolated from marine sources also showed interesting biological properties. In particular, the batzelladine alkaloids have been found to be potent HIV gp-120-CD4 inhibitors [6,7]. 

The most simple and straightforward procedure for the synthesis of DHPMs was first reported by the Italian chemist Pietro Biginelli more than 100 years ago; it involves a three-component one-pot condensation of benzaldehyde, ethyl acetoacetate and urea under strongly acidic conditions [8]. However, this reaction often requires harsh conditions and long reaction times and affords low yields, particularly when substituted aromatic and aliphatic aldehydes are employed. In recent years several methods for the synthesis of DHPMs have been developed to improve and modify this reaction by means of microwave irradiation [9], ultrasound irradiation [10], ionic liquids [11], Lewis and protic acid promoters such as lanthanide triflate [12], H_3_BO_3_ [13], VCl_3_ [14], Sr(OTf)_2_ [15], PPh_3_ [16], Indium(III) halides [17], KAl(SO_4_)_2_·12H_2_O supported on silica [18], Silicasulfuric acid [19], Mn(OAc)_3_·2H_2_O [20], Y(NO_3_)_3_·6H_2_O [21], In(OTf)_3_ [22], TaBr_5_ [23], Ce(NO_3_)_3_·6H_2_O [24], silica chloride [25], HCOOH [26] and so on. However, in spite of their potential utility, many of these reported one-pot protocols suffer from drawbacks such as the use of expensive reagents, strong acidic conditions and long reaction times. Therefore, to avoid these limitations, the introduction of a milder and more efficiently methods accompanied with higher yields are needed.

**Scheme 1:**
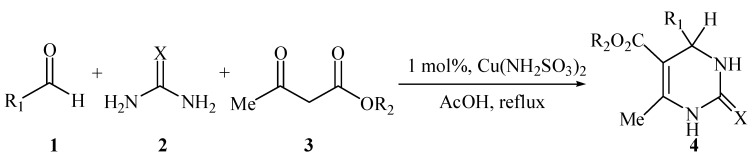
Cu(NH_2_SO_3_)_2_-catalyzed Biginelli reaction.

To our knowledge, neither copper(II) sulfamate nor its derivatives have been explored as a catalyst for any organic transformations. In continuation of our interest in Lewis acid applications for the Biginelli reaction [27,28], herein we wish to report for the first time a novel, simple and efficient methodology for the synthesis of 3,4-dihydropyrimidin-2(1*H*)-ones and thione analogs in moderate to good yields by the reaction of aldehydes, *β*-ketoesters and urea or thiourea using copper(II) sulfamate as catalyst (Scheme 1).

## Results and Discussion

To evaluate the effect of the catalyst under different conditions, the reaction of benzaldehyde, ethyl acetoacetate and urea was selected as a model reaction and the results are presented in Table 1. Initially the effect of the solvent on the reaction was studied (Table 1, entries 1–5) and glacial acetic acid was found to be the best. The amount of Cu(NH_2_SO_3_)_2_ was examined next and the results are summarized in Table 1, entries 5, 7–12. It could be seen that 1 mole% of Cu(NH_2_SO_3_)_2_ gave the best result (Table 1, entry 10), whereas in the absence of copper(II) sulfamate and under the same reaction conditions, the yield of the product **4a** was only 33% (Table 1, entry 6). The influence of the reaction time on the yield was also investigated as shown in Table 1, entries 10, 13–18. It was found that higher yield ocurred when the reaction time was 5 h. Hence, the best conditions employ 0.01:1:1:1.5 mole ratio of Cu(NH_2_SO_3_)_2_, benzaldehyde, ethyl acetoacetate and urea at 100 °C for 5 h using glacial acetic acid as solvent.

**Table 1:** Effect of catalyst Cu(NH_2_SO_3_)_2 _under different reaction conditions for condensation of benzaldehyde, ethyl acetoacetate and urea ^a^.

Entry	Solvent	Cu(NH_2_SO_3_)_2_ (mol%)	Time (h)	Yield (%)^b^
1	EtOH	2.5	6	61
2	CH_3_CN	2.5	6	52
3	H_2_O	2.5	6	16
4	Toluene	2.5	6	38
5	HAc	2.5	6	65
6	HAc	none	6	33
7	HAc	0.1	6	49
8	HAc	0.3	6	62
9	HAc	0.5	6	74
10	HAc	1.0	6	77
11	HAc	1.5	6	76
12	HAc	2.0	6	73
13	HAc	1.0	1	61
14	HAc	1.0	2	66
15	HAc	1.0	3	70
16	HAc	1.0	4	75
17	HAc	1.0	5	79
18	HAc	1.0	7	70

^a ^Reaction conditions: benzaldehyde (2 mmol), ethyl acetoacetate (2 mmol), urea (3 mmol) and catalyst in solvent (10 mL), 100 °C; ^b ^Isolated yield.

In order to study the scope of the procedure, a series of DHPMs were synthesized using the new reaction set-up. The results are listed in Table 2. In all cases studied, the three-component reaction proceeded smoothly to give the corresponding DHPMs in satisfactory yields. Most importantly, aromatic aldehydes carrying either electron donating or electron withdrawing substituents reacted very well to give the corresponding DHPMs with high purity in moderate to good yields. Another important feature of this procedure is the tolerance of various functional groups such as methoxy, halides, nitro, hydroxy, etc. to the reaction conditions, as well as the compatibility without formation of side products of acid sensitive aldehydes such as furfural and cinnamaldehyde. Thiourea has been used with similar success to provide corresponding *S*-dihydropyrimidinones analogues, which are also of interest due to their biological activities (Table 2, entries **4n**–**4p**). The use of methyl acetoacetate as 1,3-dicarbonyl moiety in place of ethyl acetoacetate also gave similar results, as shown in Table 2 (entries **4q**–**4t**).

**Table 2:** Cu(NH_2_SO_3_)_2_-catalyzed one-pot synthesis of 3,4-dihydropyrimidin-2(1*H*)-ones/thiones^a^

Entry	R_1_	R_2_	X	Yields (%)^b^	Mp (°C)^c^	
					Found	Reported (Lit.)
**4a**	C_6_H_5_	EtO	O	79	201-204	202-203[22]
**4b**	4-CH_3_O-C_6_H_4_	EtO	O	69	204-206	203-204[22]
**4c**	C_6_H_5_-CH=CH	EtO	O	84	234-236	234-236[22]
**4d**	4-F-C_6_H_4_	EtO	O	68	183-185	175-177[8]
**4e**	3-Br-C_6_H_4_	EtO	O	84	190-192	185-186[21]
**4f**	4-CH_3_-C_6_H_4_	EtO	O	79	212-213	216-217[12]
**4g**	4-Cl-C_6_H_4_	EtO	O	75	212-214	212-214[6]
**4h**	3-NO_2_-C_6_H_4_	EtO	O	89	224-226	226-228[22]
**4i**	3-CH_3_O-4-HO-C_6_H_3_	EtO	O	75	233-235	233-235[22]
**4j**	4-HO-C_6_H_4_	EtO	O	79	231-233	231-233[22]
**4k**	3,4-(CH_3_O)_2_-C_6_H_3_	EtO	O	70	175-177	175-177[15]
**4l**	2,4-(Cl)_2_-C_6_H_3_	EtO	O	91	246-248	251-252[6]
**4m**	2-Furyl	EtO	O	61	206-208	205-207[22]
**4n**	C_6_H_5_	EtO	S	65	207-209	206-208[22]
**4o**	3-NO_2_-C_6_H_4_	EtO	S	59	210-212	203-205[22]
**4p**	3-Br-C_6_H_4_	EtO	S	59	182-184	–
**4q**	C_6_H_5_	MeO	O	70	212-214	210-212[15]
**4r**	4-HO-C_6_H_4_	MeO	O	79	231-232	231-233[21]
**4s**	3-CH_3_O-4-HO-C_6_H_3_	MeO	O	82	248-250	253-254[11]
**4t**	2,4-(Cl)_2_-C_6_H_3_	MeO	O	72	256-258	254-255[8]

^a^ Reaction conditions: aldehyde (2 mmol), *β*-ketoester (2 mmol), urea or thiourea (3 mmol), Cu(NH_2_SO_3_)_2 _(0.02 mmol), HAc (10 mL), 100 °C; ^b ^Isolated yield; ^c^ Melting points are uncorrected.

**Scheme 2:**
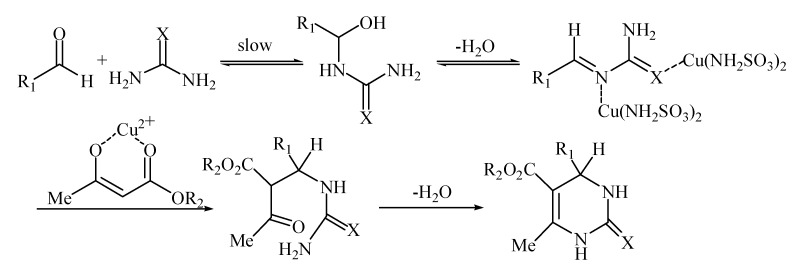
Mechanism of the Biginelli reaction catalyzed by Cu(NH_2_SO_3_)_2._

A proposed reaction mechanism for the Biginelli condensation via an acyl imine intermediate, according to the mechanism suggested by Kappe [29], is presented in Scheme 2. This intermediate is formed by the reaction of the aldehyde and urea or thiourea and then stabilized by Cu(NH_2_SO_3_)_2_ through a coordinate bond due to its empty orbital. Subsequent addition of ethyl acetoacetate enolate to the acylimine, followed by cyclization and dehydration, affords the corresponding dihydropyrimidinones.

## Conclusions

In conclusion, we have described the first time use of copper(II) sulfamate as an efficient catalyst for the synthesis of 3,4-dihydropyrimidin-2-(1*H*)-ones and thione analogs by multicomponent Biginelli reactions. The protocol offers several advantages such as mild reaction conditions, short reaction times, easy isolation and good yields. Further work is in progress to extend the catalytic activity of copper(II) sulfamate to other organic transformations.

## Experimental

### General

All compounds were characterized by IR, ^1^H NMR spectra and elemental analysis. The IR spectra were obtained as potassium bromide pellets with a FTS-40 spectrometer (Bio-Rad, U.S.A). The ^1^H NMR spectra were obtained on a Varian Inova-400 spectrometer using CDCl_3_ or DMSO-d_6_ as solvent and TMS as an internal standard, chemical shifts are given in ppm. Elemental analysis (C, H, N) was performed on a Perkin-Elmer Analyzer 2400. Melting points were determined using a Büchi B-540 instrument. All melting points are uncorrected.

### General procedure for the synthesis of 3,4-dihydropyrimidin-2(1H)-(thio)ones

A mixture of aldehyde (2 mmol), ethyl acetoacetate (2 mmol), urea or thiourea (3 mmol) and Cu(NH_2_SO_3_)_2 _(0.02 mmol) was refluxed at 100°C in glacial acetic acid (10 mL) for 5 h without stirring. The completion of the reaction was monitored by TLC. After cooling, the reaction mixture was poured onto crushed ice (50 g) and stirred for 5 min. The separated solid was filtered under suction, washed with cold water (50 mL) and then recrystallized from ethanol to afford the pure product. The results are summarized in Table 2. All products (except **4p**) are known compounds, which were characterized by mp, IR, ^1^H-NMR spectra and elemental analysis.

*5-Ethoxycarbonyl-6-methyl-4-(3-bromophenyl)-3,4-dihydropyrimidin-2(1H)-thione* (**4p**): Mp 182-184 °C; ^1^H-NMR (DMSO-d_6_) δ: 1.12 (t, 3H, *J* = 7.2 Hz, OCH_2_CH_3_), 2.30 (s, 3H, CH_3_), 4.03 (q, 2H, *J*= 7.2 Hz, OCH_2_), 5.17 (s, 1H, CH), 7.20-7.50 (m, 4H, Ar-H), 9.68 (s, 1H, NH), 10.41 (s, 1H, NH); ^13^C-NMR (DMSO-d_6_) δ: 14.64, 17.85, 54.21, 60.35, 100.75, 122.32, 126.04, 129.92, 131.22, 131.64, 146.22, 146.67, 165.59, 174.99; IR (ν*_max_*; KBr, cm^–1^): 3228, 3099, 2976, 1707, 1652, 1589, 1284, 1225, 1090, 767; Anal. calcd. (%) for C_14_H_15_N_2_O_2_S: C 61.07, H 5.49, N 10.17. Found: C 61.21, H 5.45, N 10.28.

## References

[1] Kappe C.O. (1993). 100 Years of the Biginelli dihydropyridine synthesis. Tetrahedron.

[2] Kappe C.O., Fabian W.M.F., Semones M.A. (1997). Conformational Analysis of 4-Aryl-Dihydropyrimidine Calcium Channel Modulators. A Comparison of Ab Initio, Semiempirical and X-Ray Crystallographic Studies. Tetrahedron.

[3] Kappe C.O. (2000). Recent Advances in the Biginelli Dihydropyrimidine Synthesis. New Tricks from an Old Dog. Acc. Chem. Res..

[4] Atwal K.S., Rovnyak G.C., Kimball S.D., Floyd D.M., Moreland S., Swanson B.N., Gougoutas D.Z., Schewartz J., Smillie K.M., Malley M.F. (1990). Dihydropyrimidine Calcium Channel Blockers. II. 3-Substituted-4-aryl-1,4-dihydro-6-methyl-5-pyrimidinecarboxylic Acid Esters as Potent Mimics of Dihydropyridines. J. Med. Chem..

[5] Nagarathnam D., Miao S.W., Lagu B., Chiu G., Fang J., Murali Dhar T.G., Zhang J., Tyagarajan S., Marzabadi M.R., Zhang F., Wong W.C., Sun W., Tian D., Zhang J., Wetzel J.M., Forray C., Chang R.S.L., Broten T.P., Schorn T.W., Chen T.B., O'Malley S., Ransom R.W., Schneck K., Bendesky R., Harrell C.M., Gluchowski C. (1999). Design and Synthesis of Novel α_1a_ Adrenoceptor-Selective Antagonists. 1. Structure-Activity Relationship in Dihydropyrimidinones. J. Med. Chem..

[6] Patil A.D., Kumar N.V., Kokke W.C., Bean M.F., Freyer A.J., Brossi C.D., Mai S., Truneh A., Faulkner D.J., Carte B., Breen A.L., Hertzberg R.P., Johnson R.K., Westly J.W., Potts B.C.M. (1995). Novel Alkaloids from the Sponge *Batzella* sp.: Inhibitors of HIV gpl20-Human CD4 Binding. J. Org. Chem..

[7] Snider B.B., Chen J., Patil A.D., Freyer A. (1996). Synthesis of the Tricyclic Portions of Batzelladines A, B and D. Revision of the Stereoehemistry of Batzelladines A and D. Tetrahedron Lett..

[8] Biginelli P. (1893). Gazz. Chim. Ital..

[9] Banik B.K., Reddy A.T., Datta A., Mukhopadhyay C. (2007). Microwave-induced Bismuth Nitrate-Catalyzed Synthesis of Dihydropyrimidones via Biginelli Condensation under Solventless Conditions. Tetrahedron Lett..

[10] Li J.T., Han J.F., Yang J.H., Li T.S. (2003). An Efficient Synthesis of 3,4-Dihydropyrimidin-2-ones Catalyzed by NH_2_SO_3_H under Ultrasound Irradiation. Ultrason. Sonochem..

[11] Peng J.J., Deng Y.Q. (2001). Ionic Liquids Catalyzed Biginelli Reaction under Solvent-free Conditions. Tetrahedron Lett..

[12] Ma Y., Qian C., Wang L., Yang M. (2000). Lanthanide Triflate Catalyzed Biginelli Reaction. One-Pot Synthesis of Dihydropyrimidinones under Solvent-Free Conditions. J. Org. Chem..

[13] Tu S.J., Fang F., Miao C.B., Jiang H., Feng Y.J., Shi D.Q., Wang X.S. (2003). One-Pot Synthesis of 3,4-Dihydropyrimidin-2(1*H*)-ones Using Boric Acid as Catalyst. Tetrahedron Lett..

[14] Sabitha G., Reddy G.S.K.K., Reddy K.B., Yadav J.S. (2003). Vanadium(III) Chloride Catalyzed Biginelli Condensation: Solution Phase Library Generation of Dihydropyrimidin-(2*H*)-ones. Tetrahedron Lett..

[15] Su W.K., Li J.J., Zheng Z.G., Shen Y.C. (2005). One-pot Synthesis of Dihydropyrimidiones Catalyz- ed by Strontium(II) triflate under Solvent-free Conditions. Tetrahedron Lett..

[16] Debache A., Amimour M., Belfaitah A., Rhouati S., Carboni B. (2008). A One-Pot Biginelli Synthesis of 3,4-Dihydropyrimidin-2-(1*H*)-ones/thiones Catalyzed by Triphenylphosphine as Lewis Base. Tetrahedron Lett..

[17] Fu N.Y., Yuan Y.F., Pang M.L., Wang J.T., Peppe C. (2003). Indium(III) Halides-Catalyzed Pre- paration of Ferrocenedihydropyrimidinones. J. Organomet. Chem..

[18] Azizian J., Mohammadi A.A., Karimi A.R., Mohammadizadeh M.R. (2006). KAl(SO_4_)_2_·12H_2_O Support ed on Silica Gel as a Novel Heterogeneous System Catalyzed Biginelli Reaction One-Pot Synthesis of Di-hydropyrimidinones under Solvent-free Conditions. Appl. Catal. A Gen..

[19] Salehi P., Dabiri M., Zolfigol M.A., Fard M.A.B. (2003). Silica Sulfuric Acid: An Efficient and Reusable Catalyst for the One-Pot Synthesis of 3,4-Dihydropyrimidin-2(1*H*)-ones. Tetrahedron Lett..

[20] Kumar K.A., Kasthuraiah M., Reddy C.S., Reddy C.D. (2001). Mn(OAc)_3_·2H_2_O- Mediated Three-Component, One-Pot, Condensation Reaction: An Efficient Synthesis of 4-Aryl-substituted 3,4-dihydropyrimidin-2-ones. Tetrahedron Lett..

[21] Nandurkar N.S., Bhanushali M.J., Bhor M.D., Bhanage B. M. (2007). Y(NO_3_)_3_·6H_2_O: A Novel and Reusable Catalyst for One Pot Synthesis of 3,4-Dihydropyrimidin-2(1*H*)-ones under Solvent-free Conditions. J. Mol. Catal. A Chem..

[22] Ghosh R., Maiti S., Chakraborty A. (2004). In(OTf)3-Catalysed One-Pot Synthesis of 3,4-Dihydropyrimidin-2(l*H*)-ones. J. Mol. Catal. A Chem..

[23] Ahmed N., Lier J.E.V. (2007). TaBr_5_-Catalyzed Biginelli Reaction: One-Pot Synthesis of 3,4-Dihydro- pyrimidin-2-(1*H*)-ones/thiones under Solvent-Free Conditions. Tetrahedron Lett..

[24] Adib M., Ghanbary K., Mostofi M., Ganjali M.R. (2006). Efficient Ce(NO_3_)_3_·6H_2_O-Catalyzed Solvent -Free Synthesis of 3,4-Dihydropyrimidin-2(1*H*)-ones. Molecules.

[25] Karade H.N., Sathe M., Kaushik M.P. (2007). Synthesis of 4-Aryl Substituted 3,4-Di hydro- pyrimidinones Using Silica-chloride Under Solvent Free Conditions. Molecules.

[26] Cheng J., Qi D.Y. (2007). An Efficient and Solvent-Free One-Pot Synthesis of Dihydropyrimidinones under Microwave Irradiation. Chin. Chem. Lett..

[27] Liu C.J., Wang J.D., Li Y.P. (2006). One-Pot Sythesis of 3,4-Dihydropyrimidin-2(1H)-ones Using Strontium(II) Nitrate as a Catalyst. J. Mol. Catal. A Chem..

[28] Zhang X.L., Li Y.P., Liu C.J., Wang J.D. (2006). An Efficient Synthesis of 4-Substituted pyrazolyl-3,4-dihydropyrimidin-2(1*H*)-(thio)ones Catalyzed by Mg(ClO4)2 under Ultrasound Irradiation. J. Mol. Catal. A Chem..

[29] Kappe C.O. (1997). A Reexamination of the Mechanism of the Biginelli Dihydropyrimidine Synthesis. Support for an *N*-Acyliminium Ion Intermediate. J. Org. Chem..

